# Cytopenias as Adverse Drug Reactions: A 10-Year Analysis of Reporting Structure, Rate, and Trend

**DOI:** 10.3390/ph19010014

**Published:** 2025-12-20

**Authors:** Ivana Stević, Slobodan M. Janković, Marija Mihailović, Ivana Jović, Marina Odalović, Valentina Marinković, Dragana Lakić

**Affiliations:** 1 Department of Social Pharmacy and Pharmaceutical Legislation, Faculty of Pharmacy, University of Belgrade, 11221 Belgrade, Serbia; 2 Department of Pharmacology and Toxicology, Faculty of Medical Sciences, University of Kragujevac, 34000 Kragujevac, Serbia; 3 National Pharmacovigilance Centre, Medicines and Medical Devices Agency of Serbia, 11221 Belgrade, Serbia

**Keywords:** adverse drug reaction, anemia, leucopenia, thrombocytopenia, pharmacovigilance

## Abstract

**Background/Objectives**: Underreporting is very common in drug-induced cytopenias (DICs) due to the late onset of symptoms and the need for laboratory confirmation and monitoring. This research aimed to analyze spontaneously reported adverse drug reaction (ADR) cases of leucopenia, anemia, thrombocytopenia, and total cytopenia, including their reporting structure, rate, and trend, globally (World) and in Serbia. **Methods**: Based on real-world data from VigiBase, analyses of the DIC reporting structure, rate, and trend over 10 years (2014–2023) were performed. The reporting rate was calculated and expressed as the number of reports per 1,000,000 inhabitants per year (ADR/million/year). Statistics included descriptions, a chi-square test, joinpoint analysis, and measures of variability. **Results**: Leucopenia was reported more often in Serbia compared to World (1.26 versus 0.96 reports/million/year, respectively), anemia more often in World (2.09 versus 1.75 reports/million/year), while thrombocytopenia reporting was comparable (1.83 reports/million/year globally versus 1.82 reports/million/year in Serbia). In Serbia, there was a constant increase in reporting throughout the observed period, regardless of the cytopenia type, while globally, anemia reports decreased over time. Most of the reported DICs were serious and occurred in females aged 45–64 years. In Serbia, 76.34% of DICs were reported by physicians compared to 31.72% globally. **Conclusions**: Although upward trends in DIC reporting are observed, variability in reporting between years was greater in Serbia than in World. Many measures are needed to promote the early detection of DICs, with the priority of increasing access to blood count results for all healthcare workers, including pharmacists.

## 1. Introduction

Cytopenias represent “a condition in which the number of blood cells is lower than normal”, i.e., a decrease in the number of erythrocytes, lower hemoglobin concentration levels (below 130 g/L in men and 120 g/L in women (anemia)), leukocyte counts below 4.5 × 10^9^/L (leucopenia), or platelet counts below 150 × 10^9^/L (thrombocytopenia). If all three disorders are present simultaneously, the disorder is called pancytopenia [1,2,3,4].

Factors driving cytopenias are numerous and can be hereditary or acquired [4,5]. In acquired cytopenias, there are various underlying mechanisms (e.g., effects on bone marrow, autoimmune disorders, nutritional deficiencies, viruses, alcohol, ionizing radiation, etc.), including drugs (drug-induced cytopenia—DIC) [5,6,7]. In addition to anticancer drugs, DIC may be induced by antibiotics, nonsteroidal anti-inflammatory drugs, antihypertensives, antiarrhythmics, antiepileptics, and many others [5,7,8].

The incidence of thrombocytopenia is estimated at 10/1,000,000 people per year [7], and neutropenia caused by non-anticancer drugs is estimated at 2.4–15.4 cases per million people per year, with a mortality rate of 5% [9]. Anemia caused by any drug type varies from 1 to 4 cases per million people per year [10], and its mortality rate can be from 4% to 70% [7,11].

The costs resulting from adverse drug reactions (ADRs) are estimated at 30–137 billion dollars per year in the United States [12,13]. In the literature, the cost per ADR varies between USD 65.0 and USD 12,129.90 [14], with the cost per ADR in high-income countries being 10 times higher than that in low- or middle-income countries [14]. One study estimated the cost per case of treating heparin-induced thrombocytopenia in France to be around EUR 3500 [15], while another in Germany estimated that of treating agranulocytosis and anemia at EUR 1240 and 880, respectively [16].

The satisfactory safety (benefits outweigh the risks under prescribed use) of an authorized drug is guaranteed by obtaining a marketing authorisation (MA) from the competent authorities. The safety data collected in clinical trials are an integral part of the documentation submitted to the authorities, and they are used to decide whether the drug meets the requirements related to the drug’s safety. However, safety data from clinical trials have some limitations such as controlled conditions, the number of patients in clinical trials being significantly smaller than the actual number of patients, or some ADRs are not reported due to very low incidence. After obtaining an MA, MA holders are obliged to monitor and report data related to the drug’s safety to the competent institutions and to assess the safety profile of the drug continuously [17,18,19].

Even with the existence of these obligations, it is estimated that reported ADRs only account for 6–10% of all ADR cases [20,21,22]. This is especially expressed in ADRs such as cytopenias because symptoms do not develop in the early stages and require laboratory confirmation and follow-up evaluation.

The pharmacovigilance system is defined as “a system used by an organisation to fulfil its legal tasks and responsibilities in relation to pharmacovigilance and designed to monitor the safety of authorised medicinal products and detect any change to their risk-benefit balance”. An active surveillance system refers to a continuous, planned, and structured process for collecting safety information, whereas passive surveillance depends on healthcare professionals or patients to spontaneously report ADRs [23,24,25].

There are sparse references related to the structure of reported cases, rates, or trends, globally or locally, on DICs. Trend analysis of spontaneous ADR reports is an important source of information that may be a basis for planning corrective measures within the pharmacovigilance systems. However, since the trends depend heavily on pharmacotherapeutic drug groups, it is essential to determine specific trends for cytopenias as ADRs of all drugs, including non-cytostatic drugs, which are currently missing in the medical literature [26]. The aim of this research is to analyze spontaneously reported ADR cases of leucopenia, anemia, thrombocytopenia, and total cytopenia, including their structure, reporting rate (RR), and reporting trend, globally (hereafter referred also as “World”) and in Serbia.

## 2. Results

The total number of globally reported cases of drug-induced leucopenia was 74,873, compared to 88 in Serbia. The number of reported ADR cases resulting in anemia in World was 161,631, while in Serbia, it was 122. Thrombocytopenia as an ADR was reported globally in 141,836 cases, while in Serbia, it was reported in 126 cases. Globally, during the observed period, leucopenia was most commonly reported with cisplatin, cyclophosphamide, fluorouracil, doxorubicin, and carboplatin, in comparison to Serbia, where it was associated with methotrexate, prednisone, methylprednisolone, hydroxychloroquine, and tocilizumab. For anemia, it was most commonly reported worldwide with acetylsalicylic acid, furosemide, dexamethasone, pantoprazole, atorvastatin, and levothyroxine, while in Serbia, it was associated with methotrexate, bisoprolol, cisplatin, furosemide, metformin, pantoprazole, etc. Carboplatin, cisplatin, dexamethasone, gemcitabine, and oxaliplatin were the most common drugs suspected to cause thrombocytopenia in World, while cyclophosphamide, furosemide, methotrexate, paracetamol, prednisone, and rituximab were suspected to cause it in Serbia.

### 2.1. Adverse Reaction Reporting Structure

#### 2.1.1. Leucopenia

The highest number of reported leucopenia cases was in the 45–64-year-old age group (34.19% World vs. 32.95% Serbia), and ~60% of reported cases occurred in females. In World, serious cases were reported in 45.59%, while three out of four reported cases of leucopenia in Serbia were serious. In Serbia, more than two-thirds of cases were reported by physicians (73.33%), while globally, the reporter’s qualification is unknown for half of the reports.

There was a statistically significant difference in reporting across all structure categories between Serbia and World, except for the seriousness criterion. The structure of reported leucopenia cases, as well as the differences between the World and Serbia, is provided in Table 1 and the Supplementary File.

#### 2.1.2. Anemia

In all five reporting structure categories (patient age, sex, whether the ADR was serious or not, the seriousness criteria, and the reporter’s qualification), significant differences were found between World and Serbia (*p* < 0.05). Most reported cases were serious, reported by physicians, and in female patients aged 45–64 years. Globally, most of the reported cases caused/prolonged hospitalization (44.92%), while in Serbia, they were classified as “other medically important condition” (53.49%). Pharmacists reported 10.64% of cases in World, compared to 2.36% in Serbia. Detailed results are presented in Table 1 and the Supplementary File.

#### 2.1.3. Thrombocytopenia

In both World and Serbia, the majority of reported cases were categorized as serious (>78%) and were in the category “other medically important condition”. Thrombocytopenia was mostly reported in patients in the 45–64 year age group. In Serbia, the majority were female (54.76%), contrasting World, where a higher number of reports were in male patients (49.92%). Additionally, in Serbia, 73.19% were reported by physicians, whereas in World, this figure was 37.30%. The difference in reporter qualification is noticeable when comparing World and Serbia: almost 15 times higher in World compared to Serbia for cases reported by pharmacists, at 10.65% vs. 0.72%, respectively. Other results are presented in Table 1 and the Supplementary File.

#### 2.1.4. Cytopenias in Total

Overall, cytopenias were mostly reported in female patients aged 45–64 years. In Serbia, the number of reported cases in female patients is ~10% higher than the globally (61.61% vs. 51.34%). The difference in reported cases between World and Serbia is even more noticeable for serious cases (80.65% vs. 67.72%) and especially in the seriousness criteria for the category “other medically important condition” (60.68% vs. 46.88%). The number of reported cases by physicians represents 76.34% of the total reported cases in Serbia, whereas in World, this proportion is significantly lower at 31.72% (*p* < 0.05).

### 2.2. Adverse Reaction Reporting Rate

Reporting rates were the highest for leucopenia, both in World and in Serbia. Globally, the number of drug-induced anemia cases was higher than that for thrombocytopenia, while in Serbia, it was the opposite. Results are presented in Table 2.

#### 2.2.1. Leucopenia

**Figure 1:**
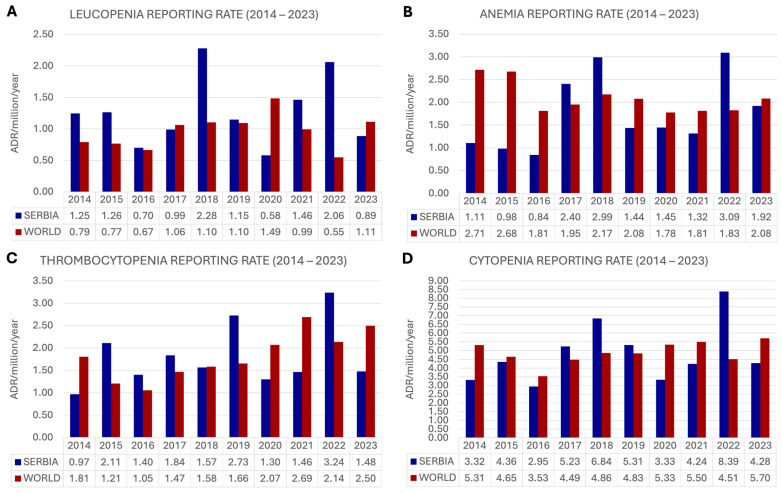
Reporting of drug-induced cytopenias from 2014 to 2023. ADR—adverse drug reaction. (**A**) Leucopenia; (**B**) Anemia; (**C**) Thrombocytopenia; (**D**) Total cytopenia.

The average reporting rate for leucopenia in World over the 10-year period was 0.96 ± 0.27 (0.55–1.49) ADR/million/year, while in Serbia, it was 1.26 ± 0.55 (0.58–2.28) ADR/million/year. Comparable RRs were observed in 2016, 2017, and 2019. In 7 out of 10 years, a higher RR was observed in Serbia. All results for the period 2014–2023 are provided in Figure 1A.

#### 2.2.2. Anemia

The average RR for anemia was higher in World compared to the rate observed in Serbia (average: 2.09 vs. 1.75 ADR/million/year). Globally, the highest RR was recorded in 2014 (2.71 ADR/million/year), while in Serbia, it was in 2022 (3.09 ADR/million/year). Conversely, the lowest RR in World was observed in 2020, while in Serbia, it was reported in 2016 (1.78 vs. 0.84 ADR/million/year, respectively). Figure 1B shows the anemia RR for World and for Serbia.

#### 2.2.3. Thrombocytopenia

For thrombocytopenia, the average RRs of World and Serbia are comparable, at 1.82 vs. 1.81 ADR/million/year. During the observed period, their RRs ranged from 0.97 to 3.24 ADR/million/year for Serbia and from 1.05 to 2.69 globally. All thrombocytopenia reporting rates are presented in Figure 1C.

#### 2.2.4. Cytopenia in Total

The reporting rate (Figure 1D) for cytopenia ranged from 2.95 ADR/million/year to 8.39 ADR/million/year in Serbia, while globally, the RR range was from 3.53 to 5.70 ADR/million/year. The average RR in World (4.87 ± 0.63, 3.53–5.70) and Serbia (4.83 ± 1.70, 2.95–8.39) over the 10-year period was comparable.

### 2.3. Adverse Reaction Reporting Trend

**Figure 2:**
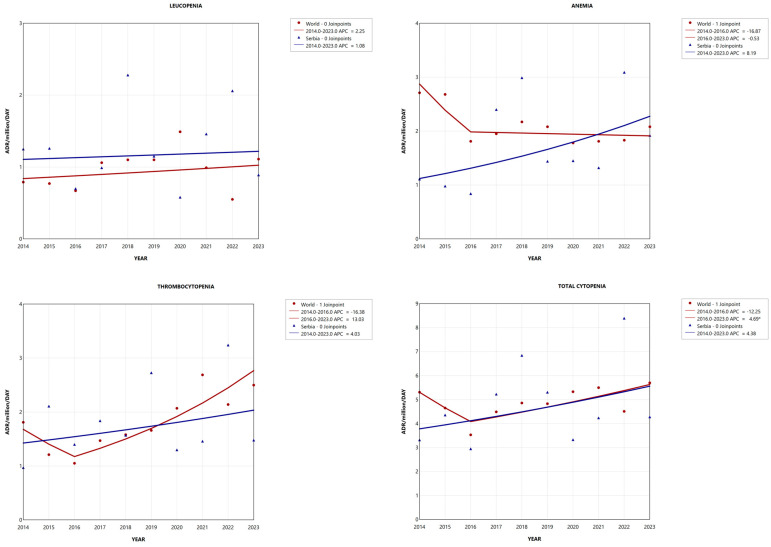
Reporting trends in drug-induced cytopenias from 2014 to 2023. ADR—adverse drug reaction; APC—annual percentage change. * Indicates that the APC is significantly different from zero at alpha = 0.05 level.

An increasing trend in the reporting of all types of DICs is observed in Serbia over a ten-year period, while in World, this trend is only evident for leucopenia. Figure 2 shows joinpoints, i.e., the points where changes in direction are observed. The variability in reporting is higher in Serbia, suggesting inconsistent pharmacovigilance practices. In the Supplementary File, additional details on trend segments and APC (annual percentage change) are provided.

#### 2.3.1. Leucopenia

Over the 10-year period, the analysis of leucopenia cases reported in World and in Serbia, expressed as ADR/million/year, showed no statistically significant change in trend (*p* > 0.05), as both showed an increase in reporting. From Figure 2, it can be seen that the growth in reporting trend was around 2 times higher in World.

The coefficient of unalikeability is greater for Serbia, indicating higher variability in the percentage change in leucopenia reporting over the years. The results are presented in Table 3.

#### 2.3.2. Anemia

Globally, the reporting trend of anemia cases showed a non-significant decrease from 2014 to 2016, with an APC of −16.87 (*p* > 0.05), and from 2016 to 2023, there was almost no change (APC = −0.53, *p* > 0.05). On the other hand, in Serbia, during this 10-year period, a continuous increase is observed throughout the reporting period, but there was also no significant change (APC = 8.19, *p* > 0.05).

The number of anemia cases reported in Serbia varied more across years; detailed information on variability is provided in Table 3.

#### 2.3.3. Thrombocytopenia

The reporting trend for thrombocytopenia showed an increase in Serbia throughout the entire observed period (APC = 4.03, *p* > 0.05). Globally, there was an increase in reported cases of thrombocytopenia from 2016 (APC = 13.03, *p* > 0.05).

The stability of the reporting process for thrombocytopenia is comparable between World and Serbia, for a 10% change in RR between years. Results for variability between years (10–50%) are presented in Table 3.

#### 2.3.4. Cytopenia in Total

The trend for the total number of cytopenia cases reported indicated an APC increase of 4.38 (*p* > 0.05) in Serbia, whereas in World, there was one joinpoint in 2016. Globally, there was a decrease before 2016 (APC = −12.25, *p* > 0.05), while an increase was seen after 2016 (APC = 4.69, *p* < 0.05). Based on Figure 2, the reporting trend for total cytopenia has been comparable between the world and Serbia since 2016.

Greater variability in the percentage change in total cytopenia reporting is observed for Serbia. In World, variability in RR between years is less than 40%, and other results are in Table 3.

## 3. Discussion

A comparison of reported cases of DIC in World and in Serbia showed that there are certain differences, specifically in the reporting of leucopenia, which is reported more often in Serbia. This is mainly because physicians are more represented as reporters and, in principle, encounter serious leucopenia often in their daily work, increasing the frequency of reports of serious leucopenia. Globally, drug-induced anemia often leads to or prolongs hospitalization, in contrast to other types of cytopenias, which most often result in “other medically important condition”, both in World and in Serbia. In Serbia, regardless of the type of cytopenia, there has been a constant increase in reporting during the observed period. On the other hand, globally, this refers to reporting leucopenia cases, while for other types of cytopenias, a joinpoint was observed, where a decrease in anemia cases has been seen in recent years. Our results show that the stability of the reporting process is higher in World, as evidenced by changes in RR percentages between years, which are lower in different types of cytopenias.

Discrepancies exist in the suspected substances reported to causes of DIC. This is not surprising, given that there are also discrepancies in treatment protocol, guidelines, accessibility, and affordability of drugs between countries. Leucopenia and thrombocytopenia were mostly reported for drugs indicated in oncology, in both World and Serbia, while for anemia, there are differences not only in substances (e.g., only furosemide is most commonly reported in both World and Serbia) but also in therapeutic groups. Regardless of the type of cytopenia and whether it is reported globally or in Serbia, the majority of cases reported occurred in women (up to 66%), except for thrombocytopenia, which was reported in 44% of females in World. These results are consistent with other publications in which the number of reported ADRs was higher in females [27,28,29,30,31,32,33,34,35,36,37]. Our result is not surprising, given that women also predominate in reports of other ADRs, possibly because they have a higher risk of developing ADRs, as shown by Rademaker [38].

For all types of analyzed cytopenias (World and Serbia), most patients were of working age, which is consistent with the results of other publications [27,29,34]. In contrast, the studies by Jiang et al. and Dubrall et al. reported the largest number of cases in patients older than 65 years [28,32]. This result is probably due to the correlation between age, the number of prescribed medications, and ADR reporting rates [39,40].

In Serbia, most cases involving any type of cytopenia, as well as total cytopenias, were reported by physicians, which aligns with other publications [32,35,36]. Globally, all types of cytopenias except leucopenia were also mostly reported by physicians; cases of leucopenia were most often reported by other healthcare workers. Pharmacists are also recognized as healthcare professionals and, depending on the healthcare system, can report ADRs [30,33,41]. In Serbia, reporting of both, all types of ADRs and DICs, comes predominantly from physicians (~60% and ~76%, respectively), while in the case of cytopenia, reporting by pharmacists is lower compared to reporting of all types of ADRs (1.69% versus 33.8%, respectively) [42]. Most reports of DICs from physicians primarily refer to patients who are already hospitalized, as they undergo more frequent blood testing, concomitantly receive more medications, and are closely monitored compared to those who are not hospitalized [43,44,45,46]. Since access to the laboratory values of patients’ results, or the ordering of laboratory analyses by pharmacists, is limited and varies between countries, it is expected that the number of reports of cytopenia as an ADR by pharmacists or patients is lower compared to that of other types of ADRs [47]. Increasing access to laboratory results for pharmacists would probably improve this situation and increase recognition and DIC reporting, as different studies imply that pharmacists’ access to laboratory results and pharmacists’ inclusion in chronic disease management lead to fewer drug-related problems, better adherence, better quality of patient care, and improved management of chronic diseases such as chronic obstructive pulmonary disease (COPD) and diabetes. In Canada, one study found that 87% of pharmacists with access to laboratory results reported better quality of care. On the other hand, Al Hamarneh et al. showed the potential contribution of pharmacists having access to laboratory results to the diagnosis of chronic diseases, with results indicating that previously undiagnosed chronic kidney diseases were detected in 40% of patients considered high risk [47,48,49,50].

Cases of ADRs characterized as serious accounted for more than 50% of reports, both globally and in Serbia, for anemia, thrombocytopenia, and total cytopenia. When reporting drug-induced leucopenia, in Serbia, 78% of cases were categorized as serious, while globally, it was less than 50%. Studies show variability in the seriousness of the different types of ADRs reported, where more than 50% of ADRs were reported as serious in some cases [32,33], while in others, less than 50% were serious [30,34,37,51]. According to the Food and Drug Administration Adverse Event Reporting System (FAERS) database, the number of reports characterized as serious is 55.17% (cut-off date: 31 March 2025) [52]. For cytopenia, more cases are reported as serious because less serious blood count disorders are very common and are often resolved without consequences; they do not paint a clear clinical picture that requires therapy or intervention from a healthcare worker, so they are often unrecognized [20,53].

Globally, the serious criterion in most reported cases was “other medically important condition” (up to 66%), except for cases of anemia, in which it was “caused/prolonged hospitalization”. It is interesting that cases of “death” varied from 4% to 7%, which is in line with other publications that refer to this category when reporting all types of ADRs [28,36]; however, according to the FAERS database, the proportion of “death cases” is a slightly higher (~9%) [52].

The RR is generally low both in World and in Serbia, which is not surprising since underreporting is recognized as a major challenge in real-world practices for all types of ADRs [20,21,22,54]. According to Stevic et al., who analyzed the costs of DICs, if the number of cases per year is compared, it can be seen that the number of cases is many times higher than the number of total annual reports for cytopenia (e.g., in Serbia, during a three-year period, 71 cases of drug-induced anemia were discovered in only one tertiary healthcare hospital after reviewing patient files, while there were only 40 reports to the National Pharmacovigilance Centre) [42,55].

The difference in RR between World and Serbia, as well as within each group, has varied over the observed period. Leucopenia and total cytopenia RRs were greater in Serbia, in contrast to anemia. For thrombocytopenia, the RR differed between years, where the RR was higher in an equal number (five) of years (in the world and in Serbia). These variations probably arise due to the variability in underreporting, which varies over time. With improved recognition of DIC and responsible reporting, these trends would probably be quite different. Additionally, it is worth noting that there are noticeable differences between countries also in treatment guidelines, the availability and accessibility of various drugs, recommendations in drug policies, and the level of reporter knowledge about pharmacovigilance and ADR reporting, which may also influence the variability in reporting [56,57,58,59].

### Limitations of the Study

Limitations and data quality related to VigiBase are also applicable to our data (e.g., suspected cases included, variation in strength of causality, heterogeneity of data, etc.) [60,61]. The analysis is limited to data from one database of spontaneous reports, where the number of cytopenia cases reported globally is compared to only one country, and due to differences in sample sizes, the effect size may indicate a small practical difference with limited clinical significance. Additionally, due to limited access to data in publicly available databases, which refer to ADR reports by drug/active substance rather than by the type of ADR, it was not possible to perform comparisons across different regions and continents.

Also, the number and type of variables present in the databases are limited, so it was not possible to analyze the causality of those differences in RR and trends, as well as the effect of confounding factors, access to healthcare, patterns of drug exposure, main drug indication, and healthcare utilization among different populations.

It should not be forgotten that the database primarily contains reports of suspected ADRs for which causality with the reported drug has not been established with certainty, so the observed trends and RRs do not reflect the actual occurrence of DIC and include reports that are subject to many factors that often cannot be identified in this type of analysis (changes in health policy, economic situation in the country, emergency situations such as COVID-19, etc.). Finally, there are differences in the age distribution of different populations, which could affect drug utilization and the number of ADRs, but this was not taken into account in this study; i.e., unlike the incidence or prevalence of a disease, reporting of ADRs does not have to be age-specific. Age standardization and systematic ascertainment bias were not considered in this type of analysis. The descriptive nature of this analysis, utilizing spontaneous reporting data, precludes the calculation of true incidence or the establishment of definitive causal relationships.

## 4. Materials and Methods

This research was designed as a secondary study based on data from spontaneously reported ADRs from the World Health Organization (WHO) global database of individual case safety reports (ICSRs)—VigiBase (VigiBase, the WHO global database of reported adverse events of medicinal products; information comes from a variety of sources, and the probability that the suspected adverse effect is drug-related is not the same in all cases (causal relationships between the event and a medicine or vaccine may be difficult to establish due to limitations in the data); information and any results and conclusions drawn do not represent the opinions of the Uppsala Monitoring Centre (UMC), the WHO Collaborating Centre for International Drug Monitoring, or the World Health Organization) [60,61]. From VigiBase, researchers from the National Pharmacovigilance Centre in Serbia independently extracted data, following data policy, on a yearly basis for the 10-year period (2014–2023) separately for the reported cases globally (referred as “World”) and for Serbia. Extracted data included the total number of case reports for leucopenia, anemia, and thrombocytopenia. Additionally, for reported cases, data were collected on the structure (distribution of ADR report cases among categories) of these cases, including patient age, sex by birth, whether the ADR was serious or not, the seriousness criteria, and the reporter’s qualification. Also, data on the 10 most commonly reported drugs (at the international non-proprietary name (INN) level) related to each cytopenia type were collected for each year. No additional inclusion/exclusion criteria were applied; the quality of the data, as well as its limitations, follows those related to VigiBase, as outlined in the caveat document and the guideline for using VigiBase data [60,61]. Reporting structure categories were for (i) age: 0–27 days, 28 days to 23 months, 2–11 years, 12–17 years, 18–44 years, 45–64 years, 65–74 years, more than 75 years, and unknown; (ii) sex by birth categories: female, male, and unknown; (iii) if the ADR is categorized as serious: yes, no, and unknown; (iv) the seriousness criteria: death, life-threatening, caused/prolonged hospitalization, disabling/incapacitating, congenital anomaly/birth defect, and other medically important conditions; and (v) the reporter’s qualification categories: physician, pharmacist, other health professionals, lawyer, consumer/non health professional, and unknown.

Based on the extracted data, IS compiled them and created datasets for leucopenia, anemia, and thrombocytopenia. Additionally, a dataset for total cytopenia was created by combining all aggregated datasets for World and for Serbia. The quality of compilation of the final dataset was independently assessed by the second researcher, SJ. Since data were not extracted at the individual patient level, but rather at the yearly level for all reports in World and Serbia, there was no coding of the data, except for coding Serbia and the World as variables.

Descriptive statistics were performed, and they included a description of the reporting structure for each cytopenia type and for total cytopenia, which was expressed as the number of reported cases (N), as well as a proportion of specific categories in the total number of reported cases (%), and it was analyzed using Microsoft Office Excel for Mac ver 16.75.

Differences in the structure of reported cases between World and Serbia were determined using the chi-square test (including Monte Carlo simulations and Fisher–Freeman–Halton Exact Test) in IBM SPSS for Mac version 29 [62]. The results were considered significant if the probability of the null hypothesis (*p*) was less than 0.05.

Data on the total number of annual reported cases for leucopenia, anemia, thrombocytopenia, and total cytopenia in World and in Serbia were used to calculate the annual reporting rate. The RR was calculated and expressed as the number of reports per 1,000,000 inhabitants per year (referred in the text as ADR/million/year). For each year, the number of inhabitants in World and in Serbia used for calculation was obtained from relevant sources [63,64].

The trend analysis, based on the reporting rate (ADR/million/year), was conducted using the joinpoint regression method, which identifies the trends between points (years) where the intensity or direction of change occurs, along with the calculation of the annual percentage change (APC). Joinpoint software version 5.4 was used to perform this analysis [65]. To evaluate the variability in reporting between years, that is, to examine the stability of the reporting process and not just the reporting trend, we also calculated the coefficient of unalikeability, where we used the degree of variability between any two years (from 10% to 50%) and assigned the categories 0 and 1 (if the difference between years was greater than or equal to the stated %, it was transformed to 1; otherwise, it was transformed to 0) [66].

## 5. Conclusions

Cytopenias are adverse drug reactions in which the clinical picture develops relatively late compared to the effect of noxa (noxae). they can also have a large number of other causes, which complicates their identification as a drug-induced disorder and thus cytopenia reporting. Although efforts are being made to increase reporting of drug-induced cytopenias, as confirmed by the upward trends revealed in our work, additional research is needed. In particular, it is important to investigate whether healthcare professionals and patients recognize adverse reactions detected through laboratory blood testing, such as cytopenias, as adverse reactions, as this would facilitate work on education and raising awareness about the importance of reporting adverse reactions. Additionally, future studies should investigate the impact of confounders (e.g., age, sex, and drug class) on reporting trends of DIC, DIC reporting for a particular drug or drug class, the causes of inconsistent pharmacovigilance practices, multinational comparisons, and the usage of causality tools and interventions to improve the reporting of ADRs. Many measures are needed, which should focus on promoting the early detection of cytopenias, primarily by increasing access to blood count results for all healthcare professionals, including pharmacists.

## Figures and Tables

**Table 1:** Total reporting structure for leucopenia, anemia, thrombocytopenia, and cytopenia in total (2014–2023) in World and Serbia.

Reporting Structure Category	World N (%)	Serbia N (%)	World N (%)	Serbia N (%)	World N (%)	Serbia N (%)	World N (%)	Serbia N (%)
ADR TYPE	Leucopenia		Anemia		Thrombocytopenia		Total Cytopenia	
**Number of reported ADR**	N = 74,873	N = 88	N = 161,631	N = 122	N = 141,836	N = 126	N = 378,340	N = 336
**PATIENT AGE**	χ^2^ = 22.606 *; ***p* = 0.003;** **w = 0.02**		χ^2^ = 29.975 *; ***p* < 0.001;** **w = 0.01**		χ^2^ = 27.381 *; ***p* < 0.001;** **w = 0.01**		χ^2^ = 63.295 *; ***p* < 0.001;** **w = 0.01**	
**Children (<18 years)**	2612 (3.48)	3 (3.41)	4534 (2.81)	2 (1.64)	6285 (4.43)	2 (1.59)	13,431 (3.54)	7 (2.08)
**Adults (18–64 years)**	37,456 (50.03)	56 (63.63)	62,840 (38.87)	74 (60.66)	61,205 (43.15)	71 (56.35)	161,501 (42.69)	201 (59.82)
**Elderly (≥65 years)**	19,630 (26.22)	10 (11.37)	60,731 (37.58)	33 (27.05)	53,846 (37.95)	29 (23.02)	134,189 (35.47)	72 (21.43)
**Unknown**	15,175 (20.27)	19 (21.59)	33,526 (20.74)	13 (10.66)	20,518 (14.47)	24 (19.05)	69,219 (18.30)	56 (16.67)
**SEX**	χ^2^ = 7.218; ***p* = 0.027;** **w = 0.01**		χ^2^ = 8.606 **; ***p* = 0.013;** **w = 0.01**		χ^2^ = 10.552 **; ***p* = 0.005;** **w = 0.01**		χ^2^ = 15.664; ***p* = 0.00;** **w = 0.01**	
**Female**	43,139 (57.62)	57 (64.77)	88,291 (54.63)	81 (66.39)	62,828 (44.29)	69 (54.76)	194,258 (51.34)	207 (61.61)
**Male**	29,388 (39.25)	25 (28.41)	64,103 (39.66)	39 (31.97)	70,816 (49.92)	56 (44.44)	164,307 (43.43)	120 (35.71)
**Unknown**	2346 (3.13)	6 (6.82)	9237 (5.71)	2 (1.64)	8201 (5.78)	1 (0.79)	19,784 (5.23)	9 (2.68)
**SERIOUS**	χ^2^ = 40.729 **; ***p* < 0.001;** **w = 0.02**		χ^2^ = 2.175 **; *p* = 0.292; **w = 0.00**		χ^2^ = 6.521 *; ***p* = 0.034;** **w = 0.00**		χ^2^ = 27.376 *; ***p* = 0.000;** **w = 0.01**	
**Yes**	34,138 (45.59)	69 (78.41)	110,393 (68.30)	91 (74.59)	111,698 (78.75)	111 (88.10)	256,229 (67.72)	271 (80.65)
**No**	40,095 (53.55)	18 (20.45)	49,651 (30.72)	30 (24.59)	28,608 (20.17)	15 (11.90)	118,354 (31.28)	63 (18.75)
**Unknown**	640 (0.85)	1 (1.14)	1587 (0.98)	1 (0.82)	1530 (1.08)	0 (0.00)	3757 (0.99)	2 (0.60)
**SERIOUSNESS CRITERIA**	χ^2^ = 2.267 *; *p* = 0.748; **w = 0.01**		χ^2^ = 11.844 **; ***p* = 0.034;** **w = 0.01**		χ^2^ = 15.371 *; ***p* = 0.008;** **w = 0.01**		χ^2^ = 29.129 **; ***p* = 0.000;** **w = 0.01**	
**Death**	1563 (3.99)	4 (4.71)	8941 (6.31)	7 (5.43)	7377 (7.22)	9 (6.57)	17,881 (6.31)	20 (5.70)
**Life threatening**	2310 (5.89)	5 (5.88)	7701 (5.43)	4 (3.10)	7853 (7.68)	6 (4.38)	17,864 (6.31)	15 (4.27)
**Caused/prolonged hospitalization**	11,312 (28.84)	21 (24.71)	63,680 (44.92)	46 (35.66)	35,395 (34.64)	31 (22.63)	110,387 (38.98)	98 (27.92)
**Disabling/incapacitating**	279 (0.71)	1 (1.18)	2440 (1.72)	2 (1.55)	1229 (1.20)	1 (0.73)	3948 (1.39)	4 (1.14)
**Congenital anomaly/birth defect**	59 (0.15)	0 (0.00)	172 (0.12)	1 (0.78)	109 (0.11)	0 (0.00)	340 (0.12)	1 (0.28)
**Other medically important condition**	23,696 (60.42)	54 (63.53)	58,825 (41.50)	69 (53.49)	50,230 (49.15)	90 (65.69)	132,751 (46.88)	213 (60.68)
**REPORTER QUALIFICATION**	χ^2^ = 190.042 *; ***p* < 0.001;** **w = 0.03**		χ^2^ = 85.014 *; ***p* < 0.001;** **w = 0.02**		χ^2^ = 122.737 *; ***p* < 0.001;** **w = 0.02**		χ^2^ = 370.128 **; ***p* < 0.001;** **w = 0.03**	
**Physician**	28,375 (15.94)	66 (73.33)	75,730 (42.72)	104 (81.89)	57,536 (37.30)	101 (73.19)	161,641 (31.72)	271 (76.34)
**Pharmacist**	9883 (5.55)	2 (2.22)	18,862 (10.64)	3 (2.36)	16,422 (10.65)	1 (0.72)	45,167 (8.86)	6 (1.69)
**Other Health Professional**	33,238 (18.67)	14 (15.56)	28,999 (16.36)	11 (8.66)	22,429 (14.54)	26 (18.84)	84,666 (16.62)	51 (14.37)
**Lawyer**	14,080 (7.91)	0 (0.00)	780 (0.44)	1 (0.79)	173 (0.11)	0 (0.00)	15,033 (2.95)	1 (0.28)
**Consumer/Non Health Professional**	3415 (1.92)	6 (6.67)	41,253 (23.27)	6 (4.72)	16,317 (10.58)	9 (6.52)	60,985 (11.97)	21 (5.92)
**Unknown**	89,052 (50.02)	2 (2.22)	11,661 (6.58)	2 (1.57)	41,357 (26.81)	1 (0.72)	142,070 (27.88)	5 (1.41)

ADR—adverse drug reaction. * Monte Carlo simulation (CI: 99%, based on 10,000 sampled tables with a starting seed of 2,000,000). ** Fisher–Freeman–Halton Exact Test. While statistically significant differences were observed (*p* < 0.05), the effect size (Cohen’s w-values) indicates a small practical difference with limited clinical significance.

**Table 2:** Reporting rates for all cytopenia types (2014–2023). Reporting rates are expressed as ADR/million/year.

ADR Type	Area	Average	Standard Deviation	Maximum	Minimum	Median	95% CI [Lower–Upper Limits]
**LEUCOPENIA**	Global	0.96	0.27	1.49	0.55	1.03	0.20 [0.77–1.16]
	Serbia	1.26	0.55	2.28	0.58	1.20	0.39 [0.87–1.66]
**ANEMIA**	Global	2.09	0.35	2.71	1.78	2.02	0.25 [1.84–2.34]
	Serbia	1.75	0.82	3.09	0.84	1.44	0.58 [1.17–2.34]
**THROMBOCYTOPENIA**	Global	1.82	0.53	2.69	1.05	1.73	0.38 [1.44–2.20]
	Serbia	1.81	0.70	3.24	0.97	1.52	0.50 [1.31–2.31]
**TOTAL CYTOPENIA**	Global	4.87	0.63	5.70	3.53	4.85	0.45 [4.42–5.32]
	Serbia	4.83	1.70	8.39	2.95	4.32	1.22 [3.61–6.04]

ADR—adverse drug reaction; CI—confidence interval.

**Table 3:** Coefficient of unalikeability for ADR reporting rates for 2014–2023.

Criteria for Unalikeability Difference *	Area	Leucopenia	Anemia	Thrombocytopenia	Total Cytopenia
**≥10%**	World	0.40	0.32	0.44	0.28
	Serbia	0.46	0.46	0.43	0.44
**≥15%**	World	0.37	0.24	0.40	0.22
	Serbia	0.41	0.43	0.38	0.42
**≥20%**	World	0.34	0.18	0.34	0.12
	Serbia	0.40	0.41	0.34	0.36
**≥25%**	World	0.31	0.13	0.29	0.08
	Serbia	0.34	0.34	0.31	0.26
**≥30%**	World	0.20	0.11	0.23	0.04
	Serbia	0.30	0.32	0.27	0.26
**≥35%**	World	0.13	0.02	0.18	0.02
	Serbia	0.28	0.29	0.19	0.22
**≥40%**	World	0.09	0.01	0.14	0.00
	Serbia	0.22	0.24	0.17	0.12
**≥45%**	World	0.08	0.01	0.08	0.00
	Serbia	0.17	0.20	0.14	0.10
**≥50%**	World	0.04	0.00	0.06	0.00
	Serbia	0.13	0.18	0.10	0.07

* The coefficient of unalikeability is calculated for different % changes in reporting rates of ADRs between any two years; if the difference between years was greater than or equal to the stated %, it was transformed to 1; otherwise, it was transformed to 0. ADR—adverse drug reaction.

## Data Availability

The original contributions presented in this study are included in the article/Supplementary Material. Further inquiries can be directed to the corresponding author.

## Supplementary Materials

The following supporting information can be downloaded at: https://www.mdpi.com/article/10.3390/ph19010014/s1, Table S1: Reporting structure for leucopenia, anemia, thrombocytopenia, and cytopenia in total (2014–2023) in the World and Serbia. Table S2: Trend segments, Annual Percent Change with 95% lower and upper limits with significance of the trend segments.
