# Supplementary material for: Cytopenias as Adverse Drug Reactions: A 10-Year Analysis of Reporting Structure, Rate, and Trend
Source: Pharmaceuticals (Basel). 2025 Dec 20;19(1):14. doi: 10.3390/ph19010014 (PMC12845036; doi:10.3390/ph19010014)
Supplement: Supplementary file 1 [file pharmaceuticals-19-00014-s001.zip › pharmaceuticals-3852207-supplementary.pdf]

Supplementary File

Table S1 Reporting structure for leucopenia, anemia, thrombocytopenia, and cytopenia in total (2014 – 2023) in the World and Serbia

**Reporting structure category**	**World N (%)**	**Serbia N (%)**	**World N (%)**	**Serbia N (%)**	**World N (%)**	**Serbia N (%)**	**World N (%)**	**Serbia N (%)**
**ADR TYPE**	**Leucopenia**		**Anemia**		**Thrombocytopenia**		**Total Cytopenia**	
**Number of reported ADR**	N = 74,873	N = 88	N = 161,631	N = 122	N = 141,836	N = 126	N = 378,340	N = 336
**PATIENT AGE**	χ2 = 22.606*; **p = 0.003; w = 0.02**		χ2 = 29.975*; **p < 0.001; w = 0.01**		χ2 = 27.381*; **p < 0.001; w = 0.01**		χ2 = 63.295*; **p < 0.001; w = 0.01**	
**0 - 27 days**	54 (0.07)	0 (0.00)	160 (0.10)	0 (0.00)	320 (0.23)	0 (0.00)	534 (0.14)	0 (0.00)
**28 days to 23 months**	302 (0.40)	0 (0.00)	877 (0.54)	1 (0.82)	1,397 (0.98)	0 (0.00)	2,576 (0.68)	1 (0.30)
**2 - 11 years**	1,096 (1.46)	1 (1.14)	1,884 (1.17)	1 (0.82)	2,522 (1.78)	1 (0.79)	5,502 (1.45)	3 (0.89)
**12 - 17 years**	1,160 (1.55)	2 (2.27)	1,613 (1.00)	0 (0.00)	2,046 (1.44)	1 (0.79)	4,819 (1.27)	3 (0.89)
**18 - 44 years**	11,860 (15.84)	27 (30.68)	21,520 (13.31)	30 (24.59)	17,262 (12.17)	19 (15.08)	50,642 (13.39)	76 (22.62)
**45 - 64 years**	25,596 (34.19)	29 (32.95)	41,320 (25.56)	44 (36.07)	43,943 (30.98)	52 (41.27)	110,859 (29.30)	125 (37.20)
**65 - 74 years**	12,561 (16.78)	9 (10.23)	28,718 (17.77)	21 (17.21)	29,879 (21.07)	25 (19.84)	71,158 (18.81)	55 (16.37)
**≥ 75 years**	7,069 (9.44)	1 (1.14)	32,013 (19.81)	12 (9.84)	23,949 (16.88)	4 (3.17)	63,031 (16.66)	17 (5.06)
**Unknown**	15,175 (20.27)	19 (21.59)	33,526 (20.74)	13 (10.66)	20,518 (14.47)	24 (19.05)	69,219 (18.30)	56 (16.67)
**SEX**	χ2 = 7.218; **p = 0.027; w = 0.01**		χ2 = 8.606**; **p = 0.013; w = 0.01**		χ2 = 10.552**; **p = 0.005; w = 0.01**		χ2 = 15.664; **p = 0.00; w = 0.01**	
**Female**	43,139 (57.62)	57 (64.77)	88,291 (54.63)	81 (66.39)	62,828 (44.29)	69 (54.76)	194,258 (51.34)	207 (61.61)
**Male**	29,388 (39.25)	25 (28.41)	64,103 (39.66)	39 (31.97)	70,816 (49.92)	56 (44.44)	164,307 (43.43)	120 (35.71)
**Unknown**	2,346 (3.13)	6 (6.82)	9,237 (5.71)	2 (1.64)	8,201 (5.78)	1 (0.79)	19,784 (5.23)	9 (2.68)
**SERIOUS**	χ2 = 40.729**; **p < 0.001; w = 0.02**		χ2 = 2.175**; p = 0.292; **w = 0.00**		χ2 = 6.521*; **p = 0.034; w = 0.00**		χ2 = 27.376*; **p = 0.000; w = 0.01**	

**Yes**	34,138 (45.59)	69 (78.41)	110,393 (68.30)	91 (74.59)	111,698 (78.75)	111 (88.10)	256,229 (67.72)	271 (80.65)
**No**	40,095 (53.55)	18 (20.45)	49,651 (30.72)	30 (24.59)	28,608 (20.17)	15 (11.90)	118,354 (31.28)	63 (18.75)
**Unknown**	640 (0.85)	1 (1.14)	1,587 (0.98)	1 (0.82)	1,530 (1.08)	0 (0.00)	3,757 (0.99)	2 (0.60)
**SERIOUSNESS CRITERIA**	χ2 = 2.267*; p =0.748**; w = 0.01**		χ2 = 11.844**; **p = 0.034; w = 0.01**		χ2 = 15.371*; **p = 0.008; w = 0.01**		χ2 = 29.129**; **p = 0.000; w = 0.01**	
**Death**	1,563 (3.99)	4 (4.71)	8,941 (6.31)	7 (5.43)	7,377 (7.22)	9 (6.57)	17,881 (6.31)	20 (5.70)
**Life threatening**	2,310 (5.89)	5 (5.88)	7,701 (5.43)	4 (3.10)	7,853 (7.68)	6 (4.38)	17,864 (6.31)	15 (4.27)
**Caused/prolonged hospitalization**	11,312 (28.84)	21 (24.71)	63,680 (44.92)	46 (35.66)	35,395 (34.64)	31 (22.63)	110,387 (38.98)	98 (27.92)
**Disabling/incapacitating**	279 (0.71)	1 (1.18)	2,440 (1.72)	2 (1.55)	1,229 (1.20)	1 (0.73)	3,948 (1.39)	4 (1.14)
**Congenital anomaly/birth defect**	59 (0.15)	0 (0.00)	172 (0.12)	1 (0.78)	109 (0.11)	0 (0.00)	340 (0.12)	1 (0.28)
**Other medically important condition**	23,696 (60.42)	54 (63.53)	58,825 (41.50)	69 (53.49)	50,230 (49.15)	90 (65.69)	132,751 (46.88)	213 (60.68)
**REPORTER QUALIFICATION**	χ2 = 190.042*; **p < 0.001; w = 0.03**		χ2 = 85.014*; **p < 0.001; w = 0.02**		χ2 = 122.737*; **p < 0.001; w = 0.02**		χ2 = 370.128**; **p < 0.001; w = 0.03**	
**Physician**	28,375 (15.94)	66 (73.33)	75,730 (42.72)	104 (81.89)	57,536 (37.30)	101 (73.19)	161,641 (31.72)	271 (76.34)
**Pharmacist**	9,883 (5.55)	2 (2.22)	18,862 (10.64)	3 (2.36)	16,422 (10.65)	1 (0.72)	45,167 (8.86)	6 (1.69)
**Other Health Professional**	33,238 (18.67)	14 (15.56)	28,999 (16.36)	11 (8.66)	22,429 (14.54)	26 (18.84)	84,666 (16.62)	51 (14.37)
**Lawyer**	14,080 (7.91)	0 (0.00)	780 (0.44)	1 (0.79)	173 (0.11)	0 (0.00)	15,033 (2.95)	1 (0.28)
**Consumer/Non Health Professional**	3,415 (1.92)	6 (6.67)	41,253 (23.27)	6 (4.72)	16,317 (10.58)	9 (6.52)	60,985 (11.97)	21 (5.92)
**Unknown**	89,052 (50.02)	2 (2.22)	11,661 (6.58)	2 (1.57)	41,357 (26.81)	1 (0.72)	142,070 (27.88)	5 (1.41)

ADR – adverse drug reaction.
* Monte Carlo simulation (CI 99%, based on 10000 sampled tables with starting seed 2,000,000)
** Fisher-Freeman-Halton Exact Test

While statistically significant differences were observed ($p<0.05$), the effect size (Cohen's w-values) indicates a small practical difference with limited clinical significance.

Table S2. Trend segments, Annual Percent Change with 95% lower and upper limits with significance of the trend segments

Adverse Drug Reaction	Area	Segment	Segment Start	Segment End	APC	APC 95% LCL	APC 95% UCL	APC Significant	p-Value
Leucopenia	World	0	2014	2023	2.2548	-4.6395	9.6351	0	0.53549
	Serbia	0	2014	2023	1.0792	-11.3505	15.0434	0	0.87543
Anemia	World	0	2014	2016	-16.87	-27.2057	0.8106	0	0.06199
	World	1	2016	2023	-0.534	-16.5916	17.0872	0	0.80784
	Serbia	0	2014	2023	8.1895	-3.9056	21.3431	0	0.19156
Thrombocytopenia	World	0	2014	2016	-16.38	-31.61	12.9353	0	0.28994
	World	1	2016	2023	13.031	-11.3618	44.6142	0	0.07119
	Serbia	0	2014	2023	4.0341	-8.7372	18.2653	0	0.49790
Total Cytopenia	World	0	2014	2016	-12.25	-21.1668	1.889	0	0.13397
	World	1	2016	2023	4.6908	0.3851	16.1518	1	**0.04679**
	Serbia	0	2014	2023	4.3806	-6.7589	16.7126	0	0.42152

APC - Annual Percentage Change; LCL - Lower Confidence Level; UCL - Upper Confidence Level
